# Cultural variation in cognitive flexibility reveals diversity in the development of executive functions

**DOI:** 10.1038/s41598-018-34756-2

**Published:** 2018-11-05

**Authors:** Cristine H. Legare, Michael T. Dale, Sarah Y. Kim, Gedeon O. Deák

**Affiliations:** 1 Department of Psychology, The University of Texas at Austin, Austin, USA; 2 Department of Cognitive Science, University of California, San Diego, La Jolla, USA

## Abstract

Cognitive flexibility, the adaptation of representations and responses to new task demands, improves dramatically in early childhood. It is unclear, however, whether flexibility is a coherent, unitary cognitive trait, or is an emergent dimension of task-specific performance that varies across populations with divergent experiences. Three- to 5-year-old English-speaking U.S. children and Tswana-speaking South African children completed two distinct language-processing cognitive flexibility tests: the FIM-Animates, a word-learning test, and the 3DCCS, a rule-switching test. U.S. and South African children did not differ in word-learning flexibility but showed similar age-related increases. In contrast, U.S. preschoolers showed an age-related increase in rule-switching flexibility but South African children did not. Verbal recall explained additional variance in both tests but did not modulate the interaction between population sample (i.e., country) and task. We hypothesize that rule-switching flexibility might be more dependent upon particular kinds of cultural experiences, whereas word-learning flexibility is less cross-culturally variable.

## Introduction

Children live in culturally-constructed niches which consist of knowledge systems, normative practices, cultural artifacts, and social institutions that vary substantially between populations. Acquiring the specific knowledge and skills of a given social groups requires a cognitive system that is highly responsive to different ontogenetic contexts and cultural ecologies^1,2^. Yet virtually all young children acquire the beliefs and practices of their social group, an extraordinary learning achievement that requires substantial ontogenetic adaptability^3–5^_._

*Flexible cognition* refers to the adaptive modification of attention, representations, and action policies in response to new task demands and ecological constraints^6^. It allows humans to build upon established behaviors by relinquishing old solutions and flexibly switching to more productive, efficient, or innovative ones^7^. It is challenging (i.e., resource-demanding) when individuals have multiple conflicting representational or behavioral options, and when they must select and integrate specific stimulus properties, task cues, and information from working and long-term memory^8,9^.

Cognitive flexibility works in tandem with other executive functions, including inhibition, attentional control, and working memory, to enable complex skills such as goal-directed planning, problem solving, and deliberate learning^10–12^. Preschool and kindergarten children’s executive function test performance is correlated with academic achievement (on both mathematical and literacy assessments)^13–15^ in the U.S., South Korea, Taiwan, and China^16–18^. There is also evidence for variation in measures of executive functioning between populations; East Asian children outperform Western children on measures of inhibition and cognitive flexibility (i.e., rule switching)^19–23^. A number of potential explanations for cultural variation in executive functioning have been proposed, including experiences in children’s home and school environments. For example, urban preschool children in China tend to receive consistent, high-demand training related to rule following and self-regulation in school and home environments^24,25^.

Cognitive flexibility, like other executive functions, improves dramatically from 3 to 6 years^26–28^. During this age span, children (from high socioeconomic status [SES] communities most widely studied in the developmental literature^2^) improve in switching between verbal rules for sorting cards^29^, in using changing semantic cues to infer novel word meanings^26^, and in other manifestations of cognitive flexibility^30–32^. This age-related pattern suggests that flexibility develops as a cohesive, general cognitive trait. The nature of this trait, however, has been a matter of debate. Age-related changes in early childhood have been attributed to representational complexity^33^, representational capacity^34,35^, cognitive inhibition^27^, attentional inhibition^36^, working memory strength^37^, and task or cue comprehension^6,38,39^.

To date, most psychological and educational research on the development of cognitive flexibility^6,10–17,21,22,26–28,32–36^ has been conducted almost exclusively in highly educated, relatively high income, highly educated, industrialized populations. The primary objective of the current study was to investigate whether cognitive flexibility develops as a coherent, unitary trait across diverse cultures, or is an emergent dimension of task-specific performance that varies between populations based on culturally-variable experiences. We investigated this in three ways. First, we compared performance on two tests of cognitive flexibility: the 3DCCS^40^, a version of the widely used Dimensional Card Change Sort Task (DCCS)^28^ and the FIM-Animates^40^, a version of the Flexible Induction of Meaning (FIM) task^26^ between two populations of 3–5-year-old children from different cultural, educational, linguistic, and SES backgrounds: a sample of English-speaking, U.S. children, and a sample of Tswana-speaking, South African children. Next, we examined the role of cue difficulty in children’s performance on each of these cognitive flexibility tests in two non-cognate languages. Finally, we assessed the impact of another potential constraint on cognitive flexibility, verbal recall, to document its relation to flexibility in rule switching and word learning in each of the populations studied. Although a cross-cultural comparison between two populations that differ on many factors cannot resolve individual causal variables, in this instance it provides an opportunity to examine whether age-related gains in flexibility, documented almost exclusively in Western, Educated, Industrialized, Rich, Democratic (WEIRD)^41^ children using only one test of flexibility, are *general* across populations and tests, or whether they differ between populations from different cultural backgrounds, and the extent to which these differences are task-specific.

Resolving the causes of age-related change in cognitive flexibility has been difficult for several reasons. First, multiple factors might contribute to the development of cognitive flexibility^42^, and they may vary both within and between populations. These may include variables strongly associated with SES (e.g., parental education, early nutrition, chronic stress), language environment (e.g., properties of first language(s); multilingualism)^43–46^, and children’s educational opportunities and experiences (e.g., activities involving symbol-mapping and rule switching). These factors may have both independent and interactive effects on the development of cognitive flexibility. Previous cross-cultural research on cognitive flexibility has been limited to comparing Western and East Asian children from relatively affluent, educated families. Educational access is strongly correlated with SES, and both vary within and between populations. This makes it difficult to test independent contributions of these factors. For example, in a meta-analysis of studies testing the relation between SES and executive functioning test performance in U.S. children^47^, greater SES-related factors (e.g., parental and educational resources) weakly but reliably predicted earlier development of executive functioning skills. Notably, the correlation between SES and executive functioning measures was strongest for “attention shifting tests,” which were defined to include rule-switching tests (a common measure of cognitive flexibility). Without examining a wider range of cultural groups and establishing cross-cultural/cross-linguistic norms for multiple kinds of flexibility tests, however, we cannot interpret the breadth or implications of such results.

Second, most research has used one cognitive flexibility test, the *Dimensional Change Card Sorting* test (DCCS), a version of intra/extradimensional reversal shift tests^48^. In this *rule-switching* test children follow instructions to switch from sorting two drawings by shape to sorting them by color, or vice versa^31^. Numerous studies using this task have shown increasing flexibility from 3 to 5 years^27,28,33,35^. In a *word-learning* test of cognitive flexibility, the Flexible Induction of Meaning paradigm (FIM)^29^, children are repeatedly shown sets of multiple complex items, and each time they are told a novel word for a ‘standard’ item. Each novel word is contextualized by a different cue phrase that implies a feature that is shared by another item is the set. The cue in each trial implies a different shared feature and therefore a different item. Flexibility is shown by generalizing each word to a different item from the set, based on the semantics of each phrase cue. In this paradigm children show robust age-related improvement from 3 to 6 years. Recent evidence from high SES children in the U.S. who completed both DCCS and FIM tasks indicate that word-learning and rule-switching flexibility might be largely dissociated: 3- to 5-year-old children showed a low correlation between measures of flexibility in the two tests^40^.

Recent findings show that high SES U.S. children’s flexibility in two FIM (word-learning) tests was only weakly associated with parallel, standardized indices of flexibility in a complexity-controlled rule-switching test (3DCCS; see below)^40^. By contrast, children’s flexibility on two word-learning tests, one with objects (FIM-Objects) and another with pictures of creatures (FIM-Animates), was strongly correlated even after controlling for age and several predictive cognitive and language indices. This suggests that in one (high SES, Western) population, word learning flexibility shows predictable age-related changes with internal consistency, but it is largely dissociated from parallel changes in rule-switching flexibility. It is currently unknown whether the dissociation is found across diverse cultural groups that differ in, for example, SES, language, and schooling. In the absence of these data, we cannot ascertain whether flexibility develops as a coherent, unitary cognitive trait^40^, or as content-specific skills that develop on relatively independent paths shaped by cultural contexts.

## Current Study

Here we investigate whether cognitive flexibility is a coherent, unitary cognitive trait, or is an emergent dimension of task-specific performance that varies across populations with culturally-variable experiences in three ways. Our first goal was to compare performance on two tests of cognitive flexibility between two populations of 3–5-year-old children from different cultural backgrounds: a sample of English-speaking, U.S. children, and a sample of Tswana-speaking, South African children. These populations differ in a number of ways including SES, language of testing and language background, and participation in academically oriented preschool. For example, the U.S. children came from higher income families than the South African children, and the U.S. parents had many more years of formal education. All of the U.S. children were currently attending preschool; the South African children had not attended preschool. The U.S. children were primarily monolingual; the South African children were all multilingual. The populations differ in other ways as well, such as diet, physical environment, and parenting belief and practices. Although a cross-cultural comparison between two populations that differ on many factors cannot resolve individual causal variables, in this instance it provides an opportunity to examine whether age-related gains in flexibility, documented almost exclusively in (WEIRD)^41^ children using only one test of flexibility, are general across tests and populations, or whether they are specific to particular cultural backgrounds, and whether the degree of generality is test-specific.

Based on previous evidence from U.S. English-learning preschool children that rule-switching and rule-learning flexibility may be divergent skills^40^, we predicted that these potentially distinct skills represent the outcomes of potentially distinct developmental processes, and therefore might show different age-related patterns across children from very different cultural backgrounds. Based on research from U.S. children, we predicted age-related changes in both word-learning and rule-switching flexibility^40^. We also predicted less cross-cultural similarity on the rule-switching test than on the word-learning test based on the hypothesis that rule-switching tests should favor WEIRD children who have more experience with formal educational activities, whereas word-learning tests should tap into language learning demands shared across all populations.

Our second goal was to increase our understanding of the role of cue difficulty in children’s performance on cognitive flexibility tests. In the Dimensional Card Change Sort Task (DCCS)^28^ children must follow changing rules to sort cards in different ways; the 3DCCS expands this by imposing switches between three rules for sorting stimuli that vary in three attributes (e.g., small yellow dog)^40^. In the Flexible Induction of Meaning (FIM) task^26^ children must use changing linguistic cues (e.g., phrase: “lives in a”) to infer meanings of new words. The FIM-Animates task tests children’s use of changing semantic information to flexibly make inductive generalizations about the meanings of different novel words for attributes of a drawing. Both tests tap into a general demand of language processing: to update representations of a speaker’s meaning by encoding and processing a ‘landscape’ of variable and changing cues. Therefore, both tests assess the ability to adapt meaning-representations to changing cues. However, the variety and forms of cues, generalizations, and response-types differ considerably. For example, in the DCCS rules and switches are arbitrary, so the test focuses on predicate logic judgments. By contrast, in the FIM the phrase cues require using conventional but diverse and unpredictable semantic implications to map new symbols to referents. Another aspect of task specificity might relate to specific semantic or conceptual content: for English-speaking children some of the particular rules or cues are easier or harder than others. Does this reflect the relative difficulty of the semantics, which might be language-specific, or does it reflect more general differences in the conceptual availability of various dimensions or meanings for children, regardless of language background?

Prior evidence shows that children do not equally readily switch to all different rules or cue. This can be seen in order effects: children are less flexible switching from an easy to a harder rule/cue than switching from a hard to an easier rule/cue^31,49,50^, as has been reported in English-speaking children^6,35,51^. However, it is unclear whether it is the *semantic implicature* of particular rules or cues that matters, or the availability of the different concepts implied by the rules or cues. This question can be addressed by testing whether these cue/rule-strength dependent order effects are language specific (i.e., tied to specific semantic implicature strength) or general (i.e., determined by the conceptual availability of various dimensions to children). Thus, if order effects reported in English speaking children replicate in our U.S. sample, but are not found in Tswana-speaking children, it will suggest a semantics based, language-specific effect. Alternatively, if order effects generalize across populations, they might indicate general conceptual biases (e.g., *species* is more intuitive than *habitat*)^52^. However, based on evidence that preschool children can readily override conceptual biases in light of language and task cues^26,53,54^, we predicted that specific semantic cue strength will determine order effects, and will therefore be language-specific.

Our third goal was to assess another constraint on the development of cognitive flexibility, and to document its relation to flexibility in each rule-switching and word-learning test for each of the populations studied. Verbal recall (VR) predicts other developing verbal processing skills in children, including comprehension and word learning^55–58^. VR differences might modulate performance on flexibility tests with verbal rules or cues. In rule-based tests, memory for the current rule might contribute to children’s ability to select the correct matching attribute; in word-learning tests, it might contribute to children’s ability to maintain a phonological representation of the novel word, the phrase cue, or both. However, we know little about the relation of VR to cognitive flexibility in children^40^, and virtually nothing about the robustness of this relation across cultures. Therefore, we assessed age differences in VR span using language-specific word and pseudo-word (i.e., non-word) lists. The goal was to determine whether VR variability in each group predicted flexibility in either test.

## Method

### Participants

Children were recruited and tested in communities in the U.S. and South Africa. All procedures in both countries were approved by the Institutional Review Board at the University of California, San Diego and by local school administrators in both communities. The study was performed in accordance with relevant ethical guidelines and regulations. Informed consent was obtained from all participants and/or their legal guardians. The U.S. sample included 60 preschool children ranging from 36 to 70 months of age (mean = 53 mo); children were uniformly distributed across that entire age range. Children were recruited and tested at non-subsidized preschools in majority English-speaking, middle SES status neighborhoods in San Diego county, California. The children were recruited and tested at licensed preschools with low teacher-child ratios, teachers with high school or college degrees, and classrooms with ample text materials (e.g., books, posters, etc.), symbolic and representational toys, and formal games that require reasoning about symbolic mappings. Teacher-lead activities often involved instructed, structured use of manipulatives and symbolic materials. Children’s spontaneous creative or text-related language efforts were, according to the preschools’ pedagogical principles, reinforced and encouraged. All children were primarily English-speaking. According to teacher reports no child had a diagnosed cognitive, language, sensory, or developmental disability. All children attended their preschool regularly and could complete the experimental tasks.

The South African (S.A.) sample included 60 Tswana-speaking preschool-aged children ranging from 36 to 73 months of age (mean = 53 mo; children were uniformly distributed across the age range). Children who were not available for two days of testing (required to complete all tasks) were not included in the data analyses. Children were recruited from a multilingual, low-SES community in a peri-urban informal settlement outside of Johannesburg in Gauteng, South Africa. Schooling in Gauteng is mandatory from 7 and 15 years, but official figures (from 1996) estimated that 11% of Black S.A. children in this age range did not attend school, and only 23% of 5-year-olds attended preschool. The Gauteng township where children were recruited is low income. Unemployment rates are high and families have limited access to resources, educational and otherwise. Children have very little access to books, toys, or preschool-educational materials, either at their homes or at the crèche where they were tested. All children in the settlement were fluent speakers of Tswana (also known as Setswana), a Bantu language of the Niger-Congo language family, and an official language of South Africa and Botswana. Tasks were translated into Tswana by bilingual research assistants and back-translated to check accuracy. Research assistants were fluent in English and Tswana. The first author of the paper was present for all data collection in South Africa and the U.S., to ensure procedural consistency.

### Materials and procedures

Each U.S. preschool child was tested individually over two sessions within a week, in a room in their preschool with distractions minimized as much as possible. Each S.A. child was tested in a community center which also serves as a daycare (crèche) for the local population. The crèche provided a safe environment and supervised social interaction with other children, but did not provide an educational curriculum or formal instruction. The flexibility tests were administered on different days. Children were randomly assigned to rule/cue orders (balanced across the age range in each sample; see below). Order of flexibility and verbal recall tests was counterbalanced. All responses by U.S. participants were scored live and were checked independently from videotapes by an independent trained observer; ambiguous scores (<1% of all responses) were resolved by discussion or corrected. Responses by the S.A. participants were coded online by two independent coders and compared *post hoc* for consistency.

#### Flexible Induction of Meaning (FIM) - Animates Test

**Figure 1:**
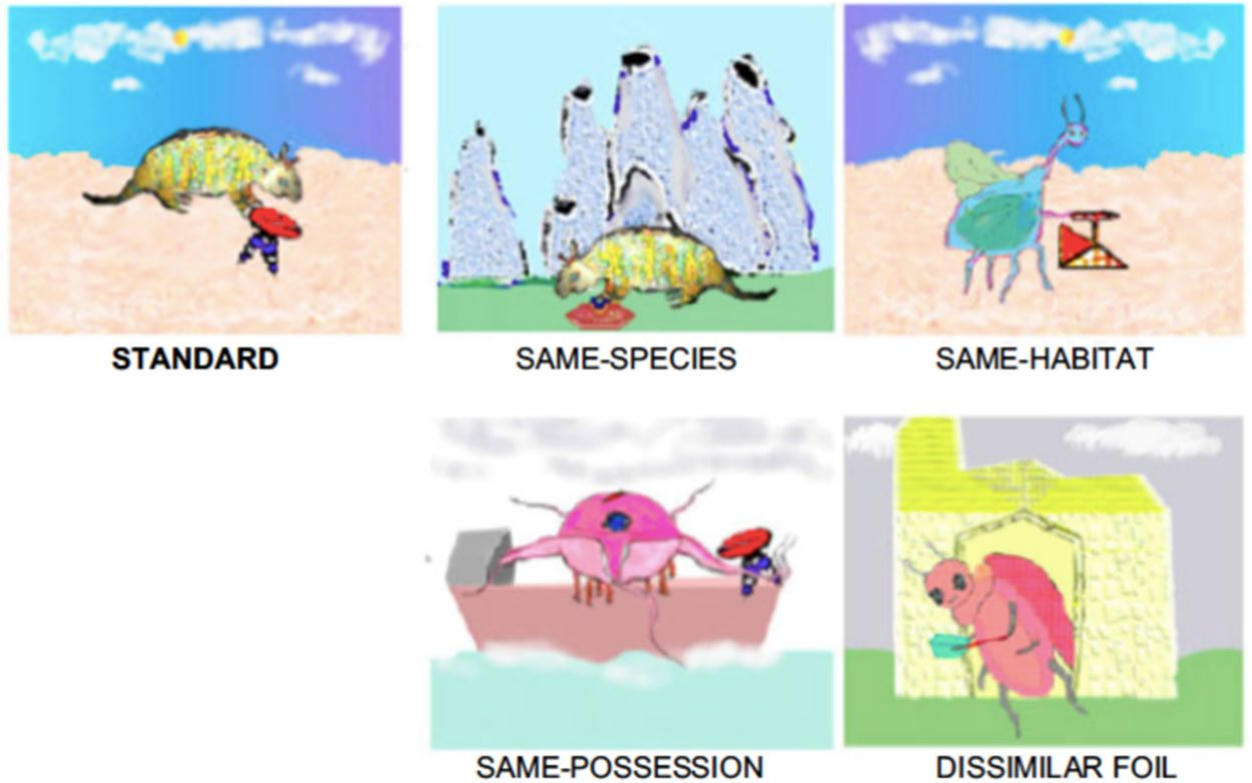
Example of a stimulus set from the FIM-An tests. Pictures in each set are clockwise from upper left: standard, same-species, same-habitat, dissimilar foil, and same-possessed-object items.

In this test children are required to infer meanings of several words for a stimulus array, based on changing sentence-level cues that imply different stimulus properties^26,51^. The stimuli include six sets of five complex color-printed and laminated pictures. Each 15 × 12.5 cm card shows a novel creature in an unfamiliar habitat, holding some distinctive object. Each set includes a ‘standard’ and four comparison pictures. Three of the comparison pictures each share one property with the standard: the species, the habitat, or the held object. The fourth comparison picture is a distractor that differs in all three properties. An example set is shown in Fig. 1.

Children completed three blocks of six trials, with one trial per set per block. Before each trial of the first block children were encouraged to examine the pictures for several seconds. The experimenter then pointed to the standard picture and told the child a fact about it. Every fact incorporated one of three predicate cues: “is a,” which (to English speakers) implies the creature’s species; “lives [in/on] a,” which implies the habitat, or “holds a,” which implies the held object. Each cue was followed by a different novel word (see Table 1). After repeating the fact (e.g., “This lives in an *oni*.”), the experimenter asked the child to generalize the word to a comparison picture (e.g., “Which of these others also lives in an *oni*”?). Children’s responses were untimed, and the experimenter repeated the fact and question again if necessary. The experimenter did not give specific feedback but made the same mildly encouraging comment after every response (“Great, let’s look at some more”). Set order was randomized for each child and the order was repeated in each block. Card positions were randomized on every trial.

**Table 1:** Novel words used in English and Tswana FIM-An Test.

Cue Semantics	Implies Species	Implies Possessed Object	Implies Habitat
English Cue	*is a…*	*holds a…*	*lives in a…*
English Novel words	leddy finnel brine ickus simee necker	minnar rett kumo dobe paydoo zyloh	toma snape volat abbick crone oni
Tswana Cue	*ke selo…*	*le leng…*	*tshela mo*
Tswana Novel words	motshelo mekgato lenong phadisano lewatle hlalosa	khudu kgokgotso anyisa goikatlisa mofufutso lefaru	sekerefolo seyalemoya malele lefaufau mosima maungo

Children were randomly assigned to a *hard* or an *easy* order, determined by results from English-speaking children^6,26,40,51^ (but of unknown difficulty for Tswana-speaking children). Children assigned to the hard order responded to the cue *is a* [or *ke solo*] in the first block of trials, *holds a* [*le leng*] in the second block, and *lives* [*in/on*] *a* [*tshela mo*] in the third block. Children in the easy condition responded to *lives* [*in/on*] *a* [*tshela mo*] in the first block, *holds a* [*le leng*] in the second block, and *is a* [*ke solo*] in the third block.

#### Translation

The first author (CL) collaborated with native Tswana-speaking educators who were familiar with the research protocols and with the children in Gauteng to develop translations of the task protocol and the cues, as well as lists of novel words that would sound natural to the children but dissimilar to known words. The protocols and materials were back-translated by different fluent bilingual adults to verify accuracy.

#### Three Dimensional Card Change Sort (3DCCS) test

**Figure 2:**
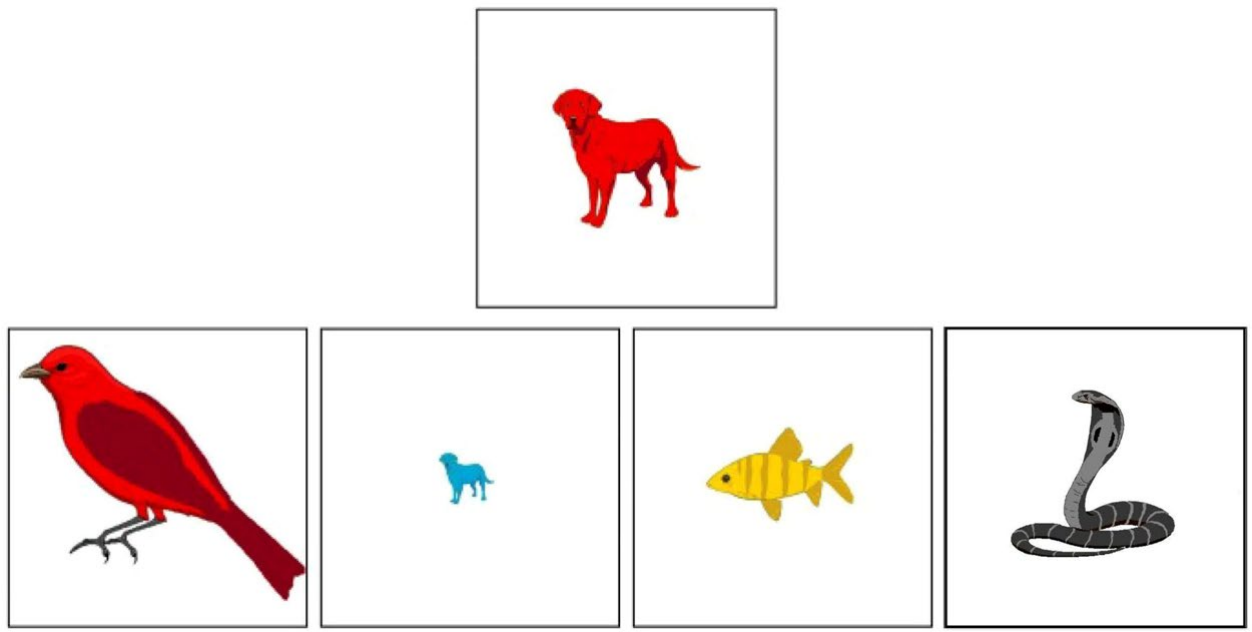
Example of a test card (top) and target cards (bottom) in the 3DCCS. The test card should be sorted into a different target box for each rule (see text).

In this test, children are asked to switch between three superordinate rules for sorting and re-sorting cards according to three dimensions: animal type, color and size. Animal types are birds, fish and dogs; colors are yellow, red and blue; and sizes are small, medium, and large. As in the FIM-An, children complete three blocks of trials, one per rule, by sorting the same six test cards, once per block. On each trial, children sort a test card into one of four boxes, each with a different target card. For example, in Fig. 2 the test card (medium red dog) should be placed in the small-blue-dog box during the animal-rule block, the large-red-bird box during the color-rule block, and the medium-yellow-fish box during the size-rule block. The fourth box (snake) is a distractor, included to check children’s attentiveness and comprehension and to equate the number of response options in the FIM-An. Each value of each dimension appeared on two test cards, but the same two values were never combined twice in the test cards seen by a given child. Test card order was randomized for each child; that order was repeated in each block. Box position was randomized on every trial.

The experimenter first checked children’s comprehension of the property labels by showing examples and asking children both to label the values of practice cards (e.g., “What animal is this”?), and to identify labeled practice cards (e.g., “Can you point to the yellow one”?). After the child showed that they could correctly label and identify all properties, the experimenter placed the boxes with standard cards in front of the child and began the test.

In each block, children were first told the current rule; for example, “In the animal game, all dogs go here, all birds go here, and all fish go here” while pointing to the relevant boxes. Then children were asked to sort each card into a box, one at a time, after hearing the picture labeled. After each trial, children received the same non-conditional feedback as in the FIM-An. After the first and second block, the experimenter reorganized the boxes on the table and gave the next instructions (e.g., “Now we are going to play the color game…”). Children were randomly assigned to one of two order conditions: *hard* or *easy*, based on results from English-speaking children (but only hypothetically easy or hard for Tswana-speaking children). The hard order was animal, color, and size. The easy order was size, color, and animal.

#### Flexibility test scoring

Flexibility in the 3DCCS and FIM-An was measured as the ratio of correct switches to the number of switching-opportunities, or CORSWOPS^6,40^. “Opportunities” are second- or third-block trials for a given stimulus set in which the cue-appropriate response (i.e., implied by the new cue) was *not* chosen by the subject in the previous trial with that set. For example, if during the *color* rule a child had previously sorted the medium/red/dog card into any box *except* the red box, then that trial would count as an *opportunity* for the child to demonstrate flexibility by switching to sort by color (which, of course, she might or might not do). Note that this discounts any trials in which the child had previously responded in a cue-inappropriate way, but that response, when repeated, became correct when the rule or cue changed. Such trials, though usually rare, are inherently ambiguous, and therefore should not be factored into measures of flexibility. CORSWOPS is therefore a more accurate, less biased estimate of flexibility. Moreover, it is comparable across tasks with different numbers of trials, blocks, or response options, so long as there is a sufficient number of opportunities to switch based on changing cues. Finally, unlike studies that classified children simply as “flexible” or “inflexible,” CORSWOPS captures not only extremes in performance (i.e., CORSWOPS approaching either 0 or 1.0), but as a scalar parameter it also captures degrees of flexibility, which are common in young children in test paradigms that are not overly simple^6,40,51^.

#### Verbal Recall (VR)

Short term verbal recall (VR) was assessed for words and non-words^55–58^. Items are shown in Table 2. The word lists included easy-to-pronounce words that would be familiar to a 3-year-old with an age-typical vocabulary. Words were randomly divided into lists of four (English) or six (Tswana) words, plus two practice lists of two words each. During pilot testing S.A. children showed ceiling effects with 4-word lists. Rather than collect a dataset invalidated by ceiling effects, the Tswana lists were made longer in order to sensitively capture individual differences in the S.A. sample without ceiling effects. Because U.S. data collection began earlier, their lists could not be made longer, but U.S. children did not show ceiling effects so such a change would not have improved the data. Non-words were constructed in each language to be easy to pronounce, distinctive, and not confusable with real words.

**Table 2:** English and Tswana words and non-words (Verbal recall).

	English Words	English Non-words	Tswana Words	Tswana Non-words
Practice	fire box	mogul jicker	mma letsogo	khurumo tladu
Practice	apple cup	nafad cam	merogo molala	leri leturi
Trial 1	peg dirt forest rug	froop kib maitai deelo	letsatsi leihlo tsela leleme marapo lelao	dilese legora lamotha mahu thoru tselo
Trial 2	muffin daisy feet table	bade geck sote chibe	tsebe lebese molomo pudi ngwana ntsa	gwai mapase kabolo mokatari mphaphathi matoto

Similar to published procedures^55,57^, the experimenter explained that after she read the words (or non-words) and said “go,” the child should repeat back as many items as she or he could remember. After completing practice lists with feedback as needed, the experimenter read each list of words, at a constant rate of 0.75 sec/item followed by the go-cue. If a child paused for more than 2 sec, the experimenter prompted them to try to remember more. All of the child’s productions were recorded and later independently coded (and verified by a senior author for accuracy); children received 1 point for each correct repetition or 0.5 points for repetitions that differed by one phoneme. To reduce proactive interference a 30 sec break was imposed after each list.

## Results

Descriptive statistics for all tasks are listed below. Table 3 includes total correct scores on the FIM-An and 3DCCS. Table 4 includes CORSWOPS for FIM-An and 3DCCS, and VR for words and non-words. Preliminary analyses indicated no gender effects. A 2 (gender) X 2 (country) ANOVA on the main measure from each test found no significant effect and only a marginal interaction with VR_word_, *F*(1,110) = 3.8, *p* = 0.055. Because two marginal results would be expected given the number of significance tests, and because the trend does not qualify our hypotheses, girls and boys were combined in all further analyses.

**Table 3:** Descriptive statistics: mean (and *SD*) of total correct responses out of 18 on the flexibility tests.

	S.A.	U.S.	All
**FIM-An**			
Easy	9.5 (3.8)	11.3 (4.0)	10.4 (4.0)
Hard	10.4 (4.4)	10.3 (4.7)	10.4 (4.6)
All	9.9 (4.1)	10.8 (4.4)	10.4 (4.2)
**3DCCS**			
Easy	8.6 (3.8)	12.4 (5.2)	10.6 (4.9)
Hard	9.0 (2.9)	12.3 (4.7)	10.5 (4.2)
All	8.8 (3.3)	12.4 (4.9)	10.6 (4.6)

**Table 4:** Descriptive statistics: Means (*SD*) of CORSWOPS (range: 0 to 1) on the flexibility tests and means of VR (SD) for words and non-words (range: 0–12 [S.A.] or 0–8 [U.S.]).

	S.A.	U.S.	All
**FIM-An**			
Easy	0.482 (0.250)	0.667 (0.407)	0.571 (0.345)
Hard	0.489 (0.309)	0.363 (0.417)	0.420 (0.374)
All	0.485 (0.275)	0.515 (0.436)	0.500 (0.365)
**3DCCS**			
Easy	0.441 (0.360)	0.622 (0.441)	0.541 (0.413)
Hard	0.373 (0.270)	0.581 (0.398)	0.472 (0.350)
All	0.404 (0.313)	0.603 (0.418)	0.506 (0.382)
**VR**			
Word	5.86 (1.52)	5.00 (2.42)	
Non-Word	2.92 (1.78)	3.30 (1.88)	

### FIM-An

**Figure 3:**
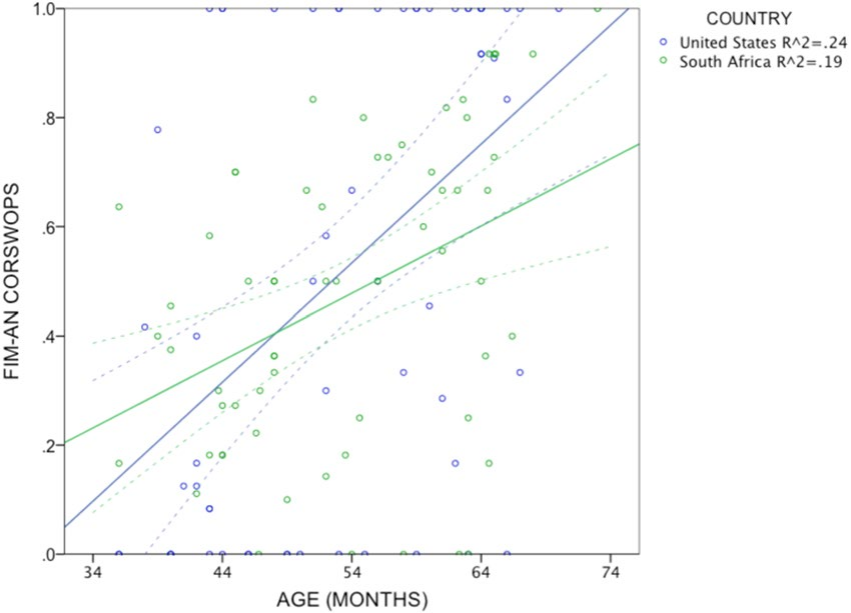
Scatterplot of FIM-An flexibility scores (CORSWOPS, or proportion of correct switches), by age, for U.S. and S.A. children. Regression lines show linear solutions, with mean confidence intervals (95%). *R*^2^ values are indicated in the legend.

Flexibility was evaluated using CORSWOPS, a measure described above that is highly correlated with total correct responses (correlations between the two measures ranged from *r* = 0.86 to 0.95 across the two tests in the two groups, *r*_mean_ = 0.89), but is more specific and unaffected by perseverative correct choices. Each child’s CORSWOPS ratio was entered in a 2 × 2 ANCOVA, with country (S.A. or U.S.) and cue order (hard or easy) between subjects, and age in months entered as a covariate. There was a significant effect of age, *F*(1,112) = 27.5, *p* < 0.001, *η*^2^_*part*_ = 0.197, but no significant effect of country (*F* < 1) (see Fig. 3). There was no significant effect of order, *F*(1,112) = 2.5, *p* = 0.116. CORSWOPS averaged 0.51 (*SD* = 0.44) for U.S. children and 0.47 (*SD* = 0.27) for S.A. children. Averages were 0.56 (*SD* = 0.35) and 0.42 (*SD* = 0.37) in the easy and the hard order, respectively. Although Levene’s test showed significant deviation from homogeneity of variance (*F*(1,118) = 8.1, *p* < 0.001), it is unlikely that this accounts for the results for several reasons: (1) A oneway ANOVA with robust means tests found that the effect of order was still significant (Welch *F*(1,114) = 4.4, *p* = 0.038); (2) heterogeneity of variance is most problematic for Type II error and when sample sizes are unequal^59^ (neither of which pertains to this case); and (3) the significant effects are fairly robust (i.e., well below the *a priori* alpha threshold).

**Figure 4:**
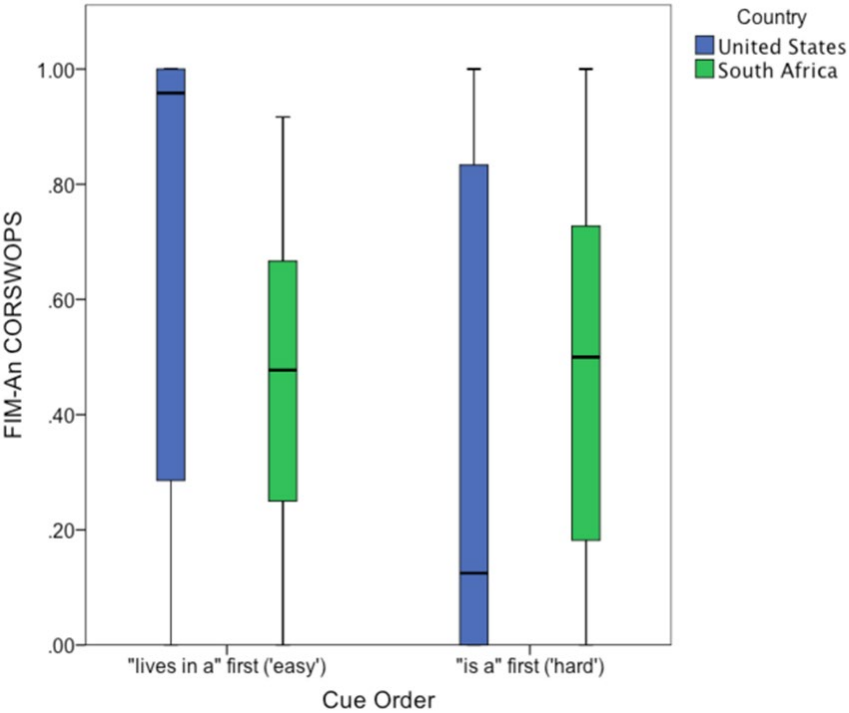
Box plot of FIM-An CORSWOPS (proportion of correct switches) by cue order (hard/*is a*-first vs. easy/*lives in*-first) in U.S. and S.A. children. Note that error bars are bounded at zero.

A significant interaction between country and order, *F*(1,105) = 8.8, *p* = 0.004, *η*^2^_*part*_ = 0.073, is illustrated in Fig. 4. U.S. children, as predicted, were less flexible when switching from the stronger (*is a*) to the weaker (*lives-in* [*a*]) cue than vice versa: CORSWOPS ($\bar{X}$) = 0.36 (*SD* = 0.42) vs. 0.67 (*SD* = 0.41). However, S.A. children showed no order effect, CORSWOPS ($\bar{X}$) = 0.49 (*SD* = 0.31) vs. 0.45 (*SD* = 0.25). This supports the speculation^6^ that semantic strength of different cues affects the difficulty of shifting cue-based inferences, so order effects will tend to be somewhat language-specific.

To further verify robustness of these results, data were re-analyzed excluding children with fewer than nine opportunities to switch, who might have skewed the CORSWOPS distribution. The results were similar for the remaining 105 (51 U.S., 54 S.A.) children, except that the order effect was significant, *F*(1,100) = 14.8, *η*^2^_*part*_ = 0.129. This effect was driven by U.S. children (means = 0.35 vs. 0.85), but not SA children (means = 0.48 vs. 0.51) and is largely consistent with the prior analysis.

To test whether age differences in flexibility differed between countries, the slopes of CORSWOPS ratios (for the entire sample) were compared for the U.S. vs. S.A. children using age as a linear regression factor. First-order statistics show that the correlation between age and CORSWOPS is similar for U.S. (*r* = 0.485) and S.A. (*r* = 0.412) children. The regression indicates that the slopes did not reliably differ between samples: *β* = 0.01 (*SE* = 0.006), *t*(113) = 1.5, *p* = 0.135.

### 3DCCS

**Figure 5:**
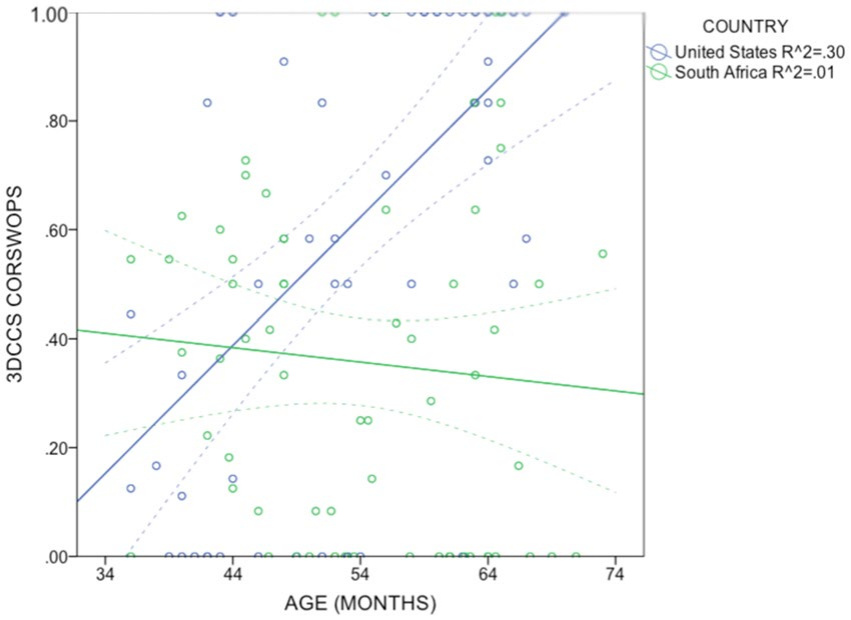
Scatterplot of 3DCCS flexibility scores (CORSWOPS, or proportion of correct switches), by age, for U.S. and S.A. children. Regression lines show linear solutions, with mean confidence intervals (95%). *R*^2^ values are indicated in the legend.

**Figure 6:**
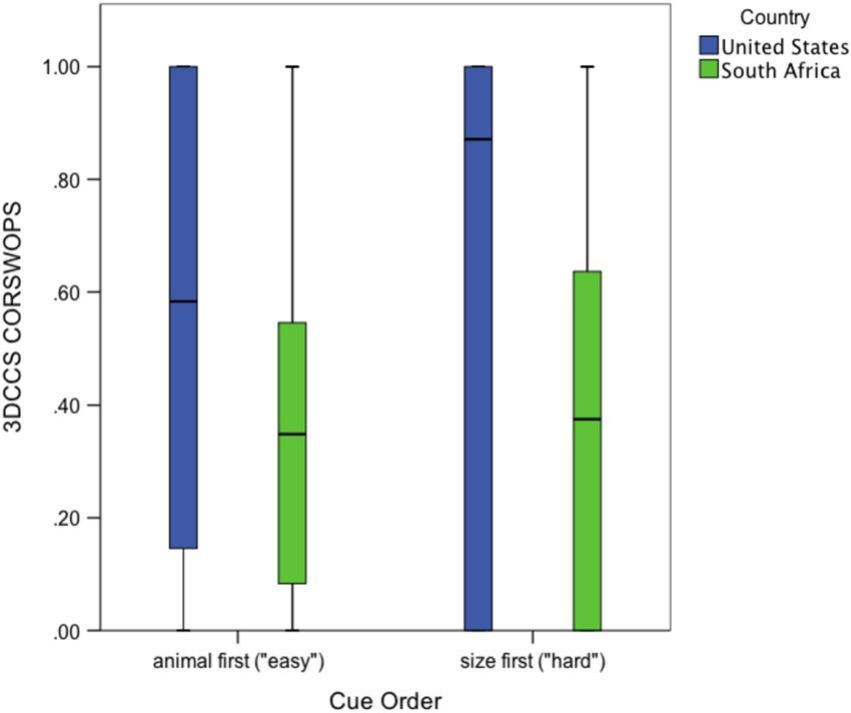
Box plot of 3DCCS CORSWOPS (proportion of correct switches) by cue order (hard order was animal, color, and size; easy order was size, color, and animal) in U.S. and S.A. children. Note that error bars are bounded at zero.

Flexibility was evaluated by entering CORSWOPS for each child into a 2 × 2 ANCOVA, with country (S.A. or U.S.) and rule order (hard or easy) between subjects, and age as a covariate (see Fig. 5). The results showed a significant age effect, *F*(1,119) = 8.8, *p* = 0.004, *η*^2^_*part*_ = 0.069 and a significant country effect, *F*(1,119) = 14.9, *p* < 0.001, *η*^2^_*part*_ = 0.111. CORSWOPS were lower for S.A. children (mean = 0.36; *SD* = 0.32) than U.S. (0.60, *SD* = 0.42). The order effect was non-significant (*F* < 1), and as was the interaction (*F* < 1): S.A. children averaged 0.38 and 0.34 for the easy and hard orders, respectively; U.S. children averaged 0.62 and 0.58; see Fig. 6. Levene’s test showed no significant deviation from homogeneity of variance (*F*(3,118) = 1.7, *ns*). A re-analysis of children who had at least 9 switch opportunities (*n* = 104), analogous to the re-analysis above, yielded very similar results.

To test whether age differences in flexibility were larger in U.S. than S.A. children, the slopes of CORSWOPS by age for U.S. and S.A. groups were compared in another linear regression. First-order statistics show a moderately high correlation between age and CORSWOPS in U.S. children (*r* = 0.546), but no significant relationship in S.A. children (*r* = 0.027). The difference between slopes is significant in the linear regression model: *β* = 0.023 (*SE* = 0.007), *t*(116) = 3.4, *p* = 0.001. The U.S. sample showed a positive association between age and rule-switching flexibility, as in previous studies, whereas the S.A. sample showed a flatter function, with no significant age effects.

### Flexibility test differences

Flexibility on the two tests was compared by calculating each child’s between-test flexibility difference, defined as [CORSWOPS_FIM_-CORSWOPS_3DCCS_]. Because we did not predict that one test would be more difficult overall, the direction of the difference is arbitrary. A between-group difference would indicate that the two tests differ in difficulty between the two groups. Differences averaged −0.09 for U.S. children (*SD* = 0.42) and +0.10 for S.A. children (*SD* = 0.35): a 19% difference in relative test difficulty. This is consistent with our prediction that the 3DCCS would be relatively harder for S.A. children. To test this pattern and its relation to age, the difference scores are entered into a univariate GLM test with nationality between-subjects and age as a covariate. The nationality effect was significant, *F*(1,117) = 6.8, *p* = 0.010, but the age covariate was not (*F*(1,117) = 1.9, *p* = 0.171). The difference score for the entire sample was not different than zero (*t*(119) < 1), confirming that the tests were similar in difficulty across the entire sample, though differentially difficult for U.S. and S.A. children.

### Verbal recall

Verbal recall (VR) was assessed separately for words and non-words in each country. Means and SDs for each sample and list type are shown in Table 4. Total recall for each list type was the sum of the two test trials totals (out of 8 or 12 per trial for U.S. and S.A. children, respectively). One U.S. and three S.A. children did not complete the non-word VR test; statistics were calculated for the remaining 116 children. Although it is not appropriate to compare raw scores between countries because the tests were not normed or standardized across cultural groups, the scores suggest that S.A. and U.S. children performed similarly.

Exploratory analyses revealed that combining total word and non-word recall yielded a more stable overall VR estimate and did not obscure any differential correlations. Further analyses therefore consider only this total (words + non-words) recall score. Additionally, because recall scores were language-specific and un-normed, total recall scores were standardized for each group. These z-scores, VR_total-std_, were correlated with age in the U.S. sample, *r*(59) = 0.49, *p* < 0.001, but uncorrelated with age in the S.A. sample, *r*(57) = −0.04.

### Predicting flexibility: Age, VR, and country

Correlations among test measures in each sample are reported in Table 5.

**Table 5:** Correlations among test measures, for each sample (top: S.A.; bottom: U.S.).

	FIM-An	3DCCS	VR_Word_	VR_Non-Word_	VR_TOTAL-Std_
**South Africa**					
Age	0.404**	−0.078	0.091	−0.143	−0.043
FIM-An		0.305* 0.327*	0.179 *0.156*	−0.034 *0.040*	0.078 *0.113*
3DCCS			0.220 *0.215*	0.310* 0.324*	0.325* 0.329*
VR_Word_				0.361** 0.379**	0.794** 0.801**
VR_Non-Word_					0.854** 0.857**
**United States**					
Age	0.485**	0.546**	0.520**	0.343**	0.488**
FIM-An		0.524** 0.350**	0.509** 0.335*	0.221 *0.072*	0.422** *0.249*
3DCCS			0.424** *0.185*	0.246* *0.102*	0.387** *0.197*
VR_Word_				0.542** 0.455**	0.905** 0.875**
VR_Non-Word_					0.848** 0.830**

FIM-An and 3DCCS: CORSWOPS. VR: items recalled (raw words, raw non-words, and z-scores of standardized combined scores). Raw correlations are shown on the top row of each line, partial correlations (controlling for age) are on the bottom row (italicized). *p < 0.05; **p < 0.01.

We tested whether variance in flexibility was predicted by verbal recall as well as age and population (i.e., country) differences, and cue/rule order. To reduce test-wise inflation of Type I error, a criterion of α < = 0.025 was adopted. With CORSWOPS scores in each test as the dependent measure, age and VR_total-std_ scores were entered in the first two steps of separate regressions (one per flexibility test). Cue order was entered in the third step, and country of origin in the fourth step.

For FIM-An CORSWOPS, age was a significant predictor in the first model, *F*(1,114) = 31.6, *p* < 0.001, *R*^2^ = 0.217. Verbal recall predicted marginal added variance in the second model, Δ*R*^2^ = 0.032, *F*_Δ_(1,113) = 11.2, *p*_ΔF_ = 0.030. Cue order predicted marginal added variance in the third step, Δ*R*^2^ = 0.027, *F*_Δ_(1,112) = 3.7, *p*_ΔF_ = 0.045. In the last step, country of origin did not explain additional variance: Δ*R*^2^ = 0.003, *F*_Δ_(1,111) < 1. Beta weights are shown in Table 6 (top). As a check of the robustness of the solution, several modified regressions were run with different verbal recall measures and orders of entry. All yielded the same results.

**Table 6:** Weights of step-wise regressions CORSWOPS in the FIM-An test (top) and the 3DCCS test (bottom), with (1) age, (2) VR_total-std_, (2) cue/rule order, and (3) country, in that order.

PREDICTOR	*β*	*SE*	*β* (standardized)	*t*	*P*
***FIM-AN***					
1. Age	0.016	0.003	0.400	4.78	<0.001
2. VR	0.073	0.030	0.199	2.39	0.019
Order	0.122	0.059	0.168	2.06	0.042
Country	−0.040	0.059	−0.055	−0.68	*ns*
***3DCCS***					
1. Age	0.011	0.003	0.261	3.10	<0.001
2. VR	0.108	0.032	0.283	3.36	0.002
3. Order	0.035	0.062	0.047	0.57	*ns*
4. Country	−0.200	0.062	−0.263	−3.21	0.002

For 3DCCS CORSWOPS, age was a significant predictor in the first model, *t* = 3.1, *p* < 0.003, *R*^2^_*adj*_ = 0.104. VR_total-std_ predicted significant added variance in the second step, Δ*R*^2^ = 0.074, *F*_Δ_(1,113) = 10.3, *p*_ΔF_ = 0.002. Rule order did not predict significant variance in the third step, Δ*R*^2^ = 0.005, *F*_Δ_(1,112) < 1. Notably, country predicted significant added variance in the final step: Δ*R*^2^ = 0.069, *F*_Δ_(1,111) = 10.3, *p*_ΔF_ = 002. Beta weights are shown in Table 6 (bottom). Several modified regressions with different VR measures and models all yielded the same results.

These results confirm and extend the previous analyses by showing that with age, VR, and cue/rule order controlled, there were no reliable cross-cultural differences in FIM-An flexibility. By contrast, there were significant cross-cultural differences in the 3DCCS.

## Discussion

The primary objective of this cross-cultural comparison was to investigate the extent to which cognitive flexibility shows a consistent pattern of development across tasks and across diverse populations, or whether it is an emergent dimension of task-specific performance that varies with culturally-dependent experiences. Children from two populations with multiple differences, including SES, languages, preschool experience^60^, completed two measures of cognitive flexibility. U.S. and South African (S.A.) 3- to 5-year-olds completed two distinct tests of cognitive flexibility, one for rule-switching (3DCCS) and one for word-learning (FIM-Animates). The English versions of these tests are similar in complexity and difficulty, but only weakly correlated for U.S. children. However, it was unknown whether S.A. children would show an analogous parallelism and independence of age differences in Tswana versions of the tests. Our data demonstrate that after controlling for verbal recall, children from both populations performed similarly on the word-learning test, showing a predicted age-related pattern of increasing flexibility. In contrast, only U.S. preschoolers, but not the S.A. children, showed the predicted age-related increase in flexibility on the rule-switching test. This result contradicts pervasive if implicit assumptions in the psychology and education literatures that age-related changes in rule-switching flexibility are general (i.e., reflect broad maturational changes in core executive functions) and universal (i.e., data from WEIRD children generalize to all children).

If South African children had been less flexible or less accurate than U.S. children on both tests of flexibility, it would have been impossible to determine which between-group differences (e.g., home environment, languages spoken, school and neighborhood differences; SES-related factors such as access to high quality education, nutrition, and health care) may explain the gap. Notably, South African and U.S. children did *not* perform differently on the word-learning flexibility task (FIM-An) or verbal recall tasks. South African children were less flexible *only* on the rule-switching task (3DCCS), and the difference was found only in older South African children: 3-year-olds in both populations were equally inflexible. Moreover, the two flexibility tests have not been found to differ significantly in difficulty or variability for U.S., English-speaking children, so there is no basis to infer that the 3DCCS is inherently harder or more sensitive than the FIM-An. The results are consistent with the hypothesis that cognitive flexibility is not a unitary cognitive trait, but rather an emergent dimension of task-specific performance that varies in trajectory based on individual children’s histories, including factors that differ systematically across cultures.

Because S.A. children performed comparably to U.S. children in word-learning flexibility and verbal recall, but *not* on the rule-switching flexibility, we can hypothesize about potential explanations for this *decalage*, or pattern of between-task differences, across groups. One potential explanation is the groups’ different preschool activities involving arbitrary, instructed changes in symbol-mappings. WEIRD children, like those in our U.S. sample, who attend well-appointed preschools with college-educated teachers typically spend considerable time in activities that impose explicit symbol-mappings, sometimes including arbitrary symbol-response mappings (e.g., participating in rule-based games and interacting with symbolic/semiotic print materials and manipulatives). Differential experience with educational activities that entail adult-dictated abstract rules with some arbitrariness of timing and/or mappings, might explain the current pattern of results: WEIRD preschool children, presumably have much more experience with this sort of arbitrary symbol-mapping exercise than children in communities with substantially less access to education and fewer educational resources. WEIRD preschool children might learn novel arbitrary symbol-mapping rules more quickly than children without formal pre-educational experience.

This hypothesis is only one possible explanation or partial explanation for the current pattern of results. The results do not allow conclusive attribution of the group differences to any single factor(s). Moreover, the hypothesis demands a more systematic characterization of the symbol-mapping activities experienced by preschool children in different cultures. Nevertheless, the hypothesis is consistent with the overall pattern of the current results, whereas many other possible explanations (e.g., differences in perinatal nutrition; language effects; familiarity with the testing materials or test procedures) cannot in any simple, obvious way explain the overall pattern of results. Our results therefore motivate future research on this topic: ideally, multi-site comparisons of performance on a battery of well-normed cognitive flexibility and ancillary tests by populations that differ in language, educational experience, and SES, supplemented by coordinated cognitive ethnographic studies to quantify differences in everyday experiences that could scaffold different flexibility skills. Relatedly, given the limited size of the current sample, it would be useful to replicate the current results with a larger sample and include additional measures of other verbal and cognitive skills that might constrain cognitive flexibility.

Our data underscore a limitation of the literature on children’s cognitive flexibility. Given that age is strongly correlated with pre-educational experience in 3- to 6-year-olds children in WEIRD populations, and the existing literature almost exclusively includes WEIRD samples, we cannot infer whether any reported age differences in flexibility are due to culture-independent processes of executive function development, or to culturally variable experiences that build specific executive functions, or both. For example, it is possible that all reported age-related changes in cognitive flexibility are due to specific skill-learning scaffolded by formal learning experiences. Alternately, it is plausible that specific experiences interact with other developmental processes and informal learning. Although the present results do not speak directly to this question, they underscore the need for research to track children receiving different culturally-specific experiences (educational or otherwise) and relate these experiences to trajectories of change in cognitive flexibility and other executive functions. Such research would begin to address how particular experiences impact the development of higher-order thinking skills.

With respect to our second goal, to examine the role of cue difficulty in performance on each cognitive flexibility test in two non-cognate languages, U.S. and South African children did not show parallel task order effects. This is consistent with the hypothesis^6^ that relative task difficulty is a function of the semantic strength of specific cues within a given task, and this variable is at least partly language-specific. That is, because children show greater costs when switching from an easier to a harder test than vice versa^31,49^, they should show less flexibility when switching from an easier sorting rule or semantic cue to a harder one. This has true of U.S. preschoolers for both the FIM-An and the 3DCCS^40,51^. However, in the current study only U.S. children, but not S.A. children, showed an order effect on the FIM-An. This suggests that order effects are based on specific cue strengths, and that phrase cues as in the FIM-An have semantic association strengths that are language-specific. Conversely, the results do not support the hypothesis that some properties are more conceptually available than others for inductive generalization, to children from a wide range of cultural and language backgrounds. In short, semantic transparency rather than conceptual availability seems to account for the relative difficulty of different verbal cues, and related phenomena such as order effects. This is consistent with evidence that task-switching flexibility depends on the transparency and familiarity of specific cues^38,39^.

With respect to our third goal, to assess the impact of verbal recall on flexibility in rule switching and word learning, the results support and extend previous findings that verbal recall (VR) capacity plays a limited role in children’s task-switching accuracy and/or speed^39,61,62^. VR, measured by word and non-word recall, was a modest positive predictor of word-meaning and rule-switching flexibility, accounting for ~3–7% of unique variance. The modest size of its contribution might explain why Deák and Wiseheart^40^ found no reliable association between the same two tests of flexibility and a different test of VR after controlling for age. However, the Memory for Names scale from the Woodcock-Johnson battery^63^, is not exclusively a VR test because it also requires visual memory and associative learning. Thus, that result is not necessarily inconsistent with the current results. Other factors might seem to mediate the relation between flexibility and VR: for example, Chevelier and colleagues^61^ found a reliable association in older preschoolers (4–5 years) but not younger preschoolers (3 years). Thus, there are outstanding questions about how and when differences in verbal working memory moderate children’s cognitive flexibility.

### Broader conclusions

Research comparing multiple age-appropriate tests is critical for understanding age, individual, and population differences in cognitive tasks^64^. Extensive reliance on a single task to measure an incredibly complex and higher-order phenotype such as cognitive flexibility is a limited research approach: it precludes or impedes analysis of the broader context of the task in question, and risks “garden path” hypotheses and assumptions. In addition, extensive reliance on samples from a single narrow cultural group also can send researchers down a garden path toward assumptions that replicated patterns are in fact human universals. These approaches, extensively repeated and self-reinforced, together limit our understanding of the development of complex cognitive capacities and their origins. The current results, by comparing just two tasks of cognitive flexibility in just two cultural groups, support the hypothesis that cognitive flexibility is an emergent dimension of task-specific performance that varies across populations with culturally-variable experiences. They thereby call into question common assumptions about cognitive flexibility development and, by extrapolation, similar assumptions about the universality of executive function development.
